# Disruption of post-thymic tolerance in skin-reactive TCR transgenic mice through the interaction of lymphopenia and intestinal microbiota

**DOI:** 10.1093/intimm/dxae018

**Published:** 2024-04-05

**Authors:** Hodaka Hayabuchi, Yukiko Tokifuji, Hayato Takahashi, Masayuki Amagai, Akihiko Yoshimura, Shunsuke Chikuma

**Affiliations:** Department of Microbiology and Immunology, Keio University School of Medicine, 35 Shinanomachi, East Lecture Hall 4F, Shinjuku, Tokyo 160-8582, Japan; Department of Microbiology and Immunology, Keio University School of Medicine, 35 Shinanomachi, East Lecture Hall 4F, Shinjuku, Tokyo 160-8582, Japan; Department of Dermatology, Keio University School of Medicine, 35 Shinanomachi, Shinjuku, Tokyo 160-8582, Japan; Department of Dermatology, Keio University School of Medicine, 35 Shinanomachi, Shinjuku, Tokyo 160-8582, Japan; Department of Microbiology and Immunology, Keio University School of Medicine, 35 Shinanomachi, East Lecture Hall 4F, Shinjuku, Tokyo 160-8582, Japan; Department of Microbiology and Immunology, Keio University School of Medicine, 35 Shinanomachi, East Lecture Hall 4F, Shinjuku, Tokyo 160-8582, Japan; Institute of Biotechnology, College of Life Sciences and Medicine, National Tsing-Hua University, 101-2 Kuang-Fu Road, Hsinchu, 300044, Taiwan

**Keywords:** animal model, autoimmunity, peripheral tolerance, psoriasis, T cell

## Abstract

Autoimmune diseases often arise from conditions where the immune system is compromised. While lymphopenia-induced proliferation (LIP) is crucial for immune system development and maturation, it is also caused by environmental insults, such as infection, and becomes a risk factor for autoimmunity in adults. We used Dsg3H1 TCR transgenic mice, whose T cells are designed to recognize desmogrein-3, a skin antigen, to explore the impact of lymphopenia on post-thymic tolerance. Dsg3H1 mice are known to delete the most highly autoreactive T cells in the thymus, and develop only subtle immune-mediated pathology in the steady state. However, we found that transient lymphopenia induced by total body irradiation (TBI) or cyclophosphamide (CY) results in massive dermatitis in Dsg3H1 mice. The symptoms included expansion and development of self-reactive T cells, their differentiation into CD44^high^ IL-17-producing helper T cells, and severe neutrophilic inflammation. Repopulation of FOXP3^+^ T regulatory cells after lymphopenia normally occurred, suggesting escape of skin-reactive conventional T cells from control by regulatory T cells. Furthermore, we found that a depletion of the intestinal microbiota by antibiotics prevents CY-induced dermatitis, indicating roles of the commensal intestinal microbiota in LIP and Th17 development *in vivo*. The current data suggested that post-thymic tolerance of Dsg3H1 mice is established on a fragile balance in the lymphoreplete immune environment and broken by the interplay between lymphopenia and intestinal microbiota. The dynamic phenotypes observed in Dsg3H1 mice prompt a re-evaluation of opportunistic lymphopenia together with the microbiota as pivotal environmental factors, impacting individuals with genetic predispositions for autoimmune diseases.

## Teaser

Lymphopenia breaks tolerance, allowing psoriasis to develop

## Introduction

Although T cells provide lifelong protection against pathogens, they can also be implicated in autoimmune diseases in certain individuals. There are multi-layered checkpoints to prevent the emergence and activation of autoreactive T cells. T-cell tolerance begins in the thymus, where strongly autoreactive T cells undergo deletion through a process known as “negative selection" (1). However, lines of evidence suggest the presence of many autoreactive T cells in the periphery, necessitating post-thymic self-tolerance (2). Molecules involved in regulatory T (Treg)-cell development and function [FOXP3 (3–6), CTLA-4 (7), CD25 (8)] and T-cell intrinsic inhibition [such as PD-1 (9) and PTPN22 (10)] play crucial roles in this process. Genetic deficiency, polymorphisms, or single nucleotide polymorphisms (SNPs) in these molecules contribute to autoimmune diseases in humans (4, 11–14) providing essential genetic factors.

In addition to genetics, environmental influences play a critical role in understanding the mechanisms underlying the maintenance and disruption of self-tolerance. Lymphopenia-induced proliferation (LIP) of T cells is recognized as a crucial factor for populating the peripheral immune system during the neonatal period (15). LIP is triggered by self-MHC/peptide ligands and the cytokine IL-7 (16), which is crucial for the neonatal immune system’s maturation (15). While essential in early life, LIP becomes an important factor in adulthood. In adults, lymphopenia is opportunistically caused by various conditions such as viral infections (17), human immunodeficiency virus (HIV), stress, immunosuppressant usage, or irradiation, prompting LIP to facilitate the repopulation of the T-cell compartment; however, it creates a risk for autoimmunity (18). In mouse models, the adoptive transfer of autoreactive T cells into recipients lacking T cells (such as Rag KO, SCID, and CD3eKO) leads to the development of autoimmune diseases (19). Additionally, in autoimmune-prone mice, transient lymphopenia triggers tissue-specific autoimmunity (20, 21). T cells undergoing LIP in adulthood not only support the proliferation of self-reactive T cells but also contribute to their differentiation into effector-like T cells (15, 22). Together, while LIP is indispensable in early life, LIP in adulthood is potentially detrimental in the context of autoimmune diseases.

Dsg3H1 TCR transgenic mice were generated to investigate the pathogenesis of pemphigus vulgaris, where the defined autoantigen is desmoglein-3 (DSG3) [reviewed in (23, 24)]. Previous studies revealed that Dsg3H1 T cells show high affinity to Dsg3-derived peptide in the context of MHC Class II, and partially undergo deletion in the thymus, in a manner dependent on DSG3 expression (25). However, the residual thymocytes populate the CD4^+^ population in the spleen and lymph nodes of the mice, containing 30%–40% TCRVβ6^+^ cells derived from the TCR transgene (as compared to 8%–10% observed in non-transgenic wildtype C57BL/6 mice) (25). Therefore, although Dsg3H1 cells are partially deleted in the thymus, skin-reactive cells could be detected in the periphery, escaping thymic tolerance becuase of endogenous TCRα rearrangement. In support of this idea, the transfer of Dsg3H1 T cells into syngeneic lymphopenic recipients reconstitutes skin pathology in multiple situations (25–28). Despite the strong autoreactivity of T cells, donor Dsg3H1 mice are almost healthy with subtle skin inflammation only in limited areas (25). Thus, we found that Dsg3H1 mice serve as a unique model to elucidate the mechanisms of post-thymic T-cell tolerance. Interestingly, idiopathic CD4 lymphocytopenia and HIV-induced immunodeficiency are associated with psoriasis (29–33), which implicates lymphopenia as a trigger of autoimmune dermatitis.

In this study, we demonstrate that the induction of lymphopenia with either total body irradiation (TBI) or the immunosuppressant cyclophosphamide (CY) provokes massive autoimmunity in Dsg3H1 mice. Additionally, we provide evidence that supports LIP and differentiation of autoreactive T cells into Th17 cells are driven by the intestinal microbiota and discuss the underlying mechanisms.

## Methods

### Animals

DsgH1 TCR Tg were generated in the previous study (25) and bred to CD45.1 congenic mice (RIKEN Bioresource Center, Japan, Strain #:RBRC00126). Wildtype C57BL/6N were from Sankyo Lab (Tokyo, Japan). IL-17a-eGFP reporter mice were as described (34). All mice in this study are on the C57BL/6 background and maintained in specific pathogen-free facilities at Keio University or Kyudo Co. LTD. (Saga, Japan). All animal experiments were approved by and carried out under the guidelines of the Animal Ethics Committee of Keio University (#A2022-324).

### Adoptive transfer model of dermatitis

The Th17-mediated dermatitis model was described in (28). Briefly, Lymph node CD4^+^ cells from Dsg3H1 CD45.1 mice were cultured in a 24-well tissue culture plate (Corning) coated with anti-CD3 and anti-CD28 mAbs (2 mg/ml each; BioLegend) at a density of 2 × 10^5^/ml in 1 ml of T-cell culture media (RPMI medium [Nacalai Tesque, Japan] supplemented with 10% fetal calf serum [Gibco], penicillin/streptomycin [Nacalai], non-essential amino acid solution [Nacalai], HEPES solution [Nacalai], sodium pyruvate solution [Nacalai], and 55 μM 2-mercaptoethanol [Gibco]). To induce pathogenic Th17 cells, mouse IL-6 (20 ng/ml: Peprotech), mouse IL-23 (20 ng/ml), human TGF-β (2 ng), mouse IL-1β (10 ng/ml), anti-mouse IFN-γ (5 mg/ml), and anti-mouse IL-4 (5 mg/ml) were added to the culture. Three days later, cells were harvested from the plate and cultured in the presence of mouse IL-23 (20 ng/ml) and mouse IL-2 (20 ng/ml) for another 3 days. On the day of transfer, 4–5 million expanded T cells were resuspended in phosphate-buffered saline (PBS) and intravenously injected into 5Gy-irradiated C57BL/6 mice.

### TBI and bone marrow transplantation

Mice were irradiated using CellRad + (PRECISION, USA) at a single dose of 8Gy. To rescue from bone marrow destruction, mice were given bone marrow from Rag2 knockout mice. Briefly, bone marrow cells were obtained by flushing the femur. After red blood cell lysis, 1 × 10^6^ bone marrow cells were intravenously injected to irradiated recipient mice. Body weight and the development of dermatitis were monitored every 2 days.

### CY treatment

8–10 weeks old mice were treated with CY (Tokyo Chemical Industry Co., Tokyo Japan cat# C2236) at day 0 and day 14. CY was first dissolved in PBS at 20 mg/ml and intraperitoneally injected to each mouse (200 mg/kg body weight). Beginning from the day of treatment, mice were monitored every 1–2 days for the development of obvious skin symptoms (redness, ulcer, eczema, cracking, and flaking).

### Cell isolation from ear skin

Pinnae of sacrificed mice were cut into pieces and digested in RPMI 10% containing FCS, 2 mg/ml collagenase D (Roche), 1.2 mg/ml hyaluronidase (Fujifilm-Wako Pure Chemicals), and 100 μg/ml DNase-I (Roche) at 37°C for 1 h. Single cells were prepared by filtering cell mixture through a 100 mm cell strainer (Greiner).

### FACS

T cells in the lymph nodes were analysed by FACS CANTOII (Becton) by a standard procedure. Fluorophore-conjugated antibodies used in this study were FITC-CD25 (Biolegend #102005), PE-TCR Vb6 (Biolegend #140004), PerCP-Cy5.5 CD4 (Thermo Fisher #45-0042-82), APC CD8a (Thermo Fisher #17-0081-82), CD44 APC (Biolegend #103012).

### Histology

Skin samples were fixed in phosphate-buffered 4% paraformaldehyde for 24 h, and then kept in 70% ethanol. Paraffin sectioning and Haematoxylin and Eosin (H&E) staining were done by a standard method.

### Antibiotics treatment

A water solution containing ampicillin (6.7 mg/ml: Nacalai Tesque Japan, # 19769-64), neomycin (6.7 mg/ml: Fujifilm-Wako, Japan, # 146-08871), vancomycin (3.3 mg/ml: Fujifilm-Wako, # 226-01306), metronidazole (6.7 mg/ml: Sigma #M3761) was prepared and kept frozen at −20°C until the time of treatment. Mice were given 0.2 ml of the solution by oral gavage every 3 days during the experimental period, starting 10 days before induction of dermatitis. In some experiments, each drug was given independently to the mice. Polymyxin B sulfate powder (Pfizer Japan) was given in drinking water at 100 mg/l.

## Results

### Dsg3H1 T cells survive only in lymphopenic recipients

**Figure 1.**
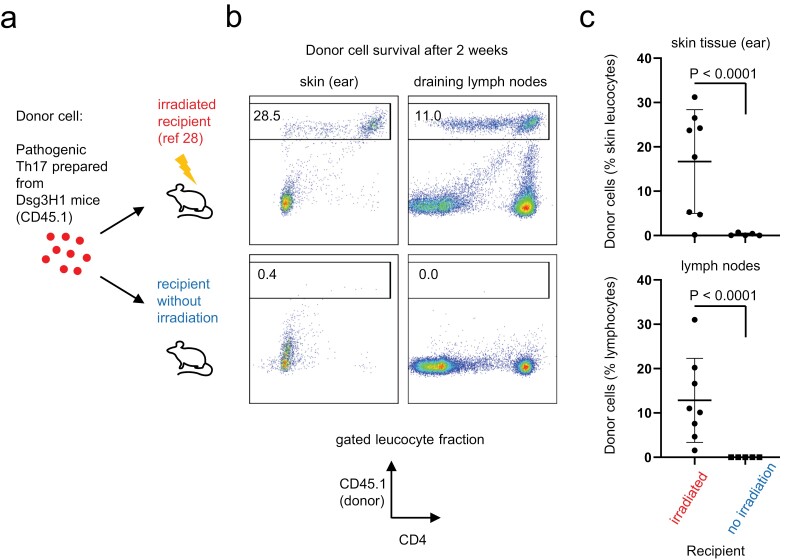
Pathogenic DsgH1 T cells survive only in irradiated recipients. (a) A scheme of experimental autoimmune dermatitis. Pathogenic Th17 cells were prepared from Dsg3H1 T cells (CD45.1 congenic) *in vivo*, as described in ref (28). The cells were transferred into either untreated or irradiated wildtype recipients (CD45.2). (b) After 2 weeks, the survival of the donor T cells was analysed based on CD45.1 expression. (c) Summary of the survived donor cells in the skin and the draining lymph nodes. Cumulative data from three independent experiments were shown. Recipients were analysed between 2 and 5 weeks after the transfer (*n* = 8 vs. 5, two-tailed *t*-test).

We recently reported that CD4^+^ T cells isolated from Dsg3H1 mice and artificially skewed into highly pathogenic Th17 (Dsg3H1-pTh17) cells *in vitro* caused chronic dermatitis upon transfer into sub-lethally irradiated wildtype recipients [(28) and Fig. 1a upper]. During the study, we attempted to examine if the transfer of Dsg3H1-pTh17 into non-irradiated, lymphoreplete recipients survived and caused dermatitis (Fig. 1a lower). However, non-irradiated recipients did not show any obvious dermatitis [(28) and unpublished data]. We found that almost all donor Dsg3H1 T cells were absent in the skin tissue (Fig 1b left panels and c), lymph nodes (Fig 1b right panels and c), or spleen (unpublished data) of the recipients. Previous studies also suggested that Dsg3H1 T cells caused dermatitis only when adoptively transferred into lymphopenic mice (25–27). Our data, together with these studies, confirmed that lymphopenia is required for the survival of moderately to strongly autoreactive T cells in Dsg3H1 mice.

### TBI induces rapid and severe dermatitis in Dsg3H1 mice

**Figure 2.**
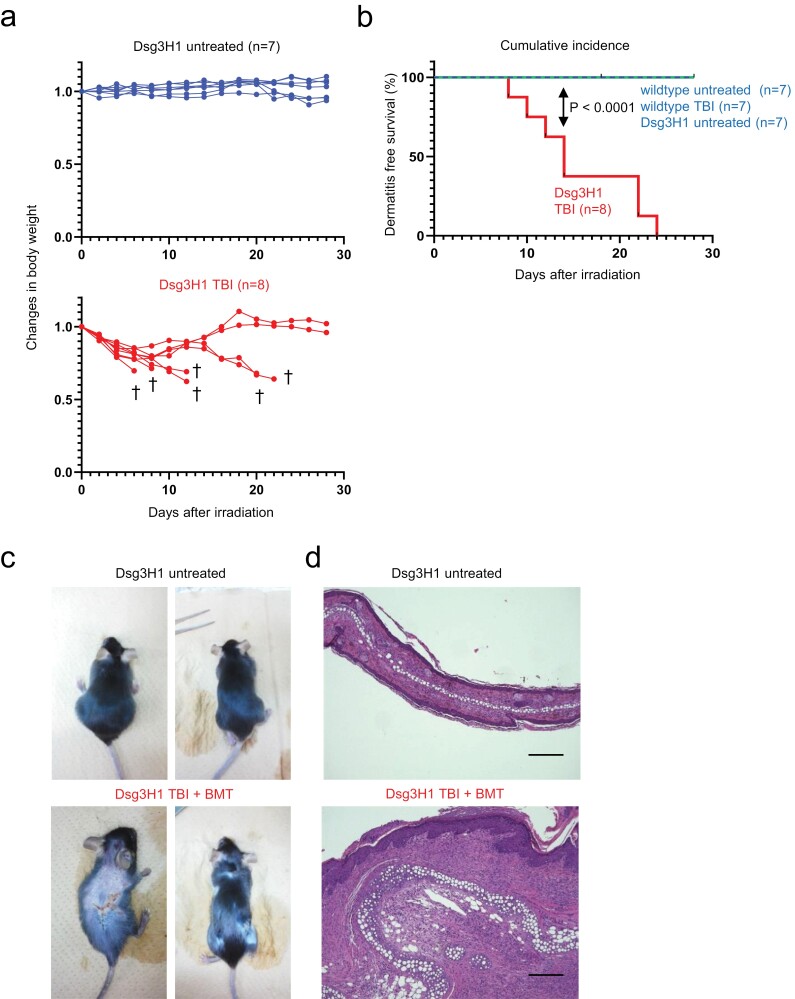
Total body irradiation (TBI) induces dermatitis in Dsg3H1 mice. Dsg3H1 mice were either untreated or given TBI (8Gy). The irradiated mice were bone marrow transplanted (BMT) to avoid death from BM destruction. (a) Changes of body weight after the treatment. Daggers (†) indicate the death of mice. (b) Cumulative incidence of death and dermatitis. The numbers of mice in each group are indicated in the panel. Log rank test (*P* < .0001). The data are representable of three independent experiments. (c, d) Macroscopic (c) and microscopic (d) dermatitis of the mice. The scale bars in (d): 200 mm.

While conducting adoptive transfer experiments (28), we noted that the donor Dsg3H1 mice developed no or only mild inflammation. We were curious if creating a transiently lymphopenic condition breaks tolerance in these mice. We treated mice with TBI, which depletes peripheral lymphocytes, followed by reconstitution by LIP (35). We found that the mice undergoing TBI showed dramatic symptoms, including weight loss, wasting, fatigue, and death (Fig 2a and b). In addition, the surviving mice developed ulcerative dermatitis on various parts of the body (Fig 2c). Histologically, the affected skin showed epidermal and dermal thickening, parakeratosis, and micro-abscesses (Fig 2d) that were similarly observed in our previous work (27, 28). Such symptoms were not observed in wildtype C57BL/6N mice with or without irradiation, or untreated Dsg3H1 mice (Fig 2b). This experiment suggested that lymphopenia by TBI triggered rapid and severe pathology in Dsg3H1 mice.

### Immune-suppressant CY induces dermatitis in Dsg3H1 mice

**Figure 3.**
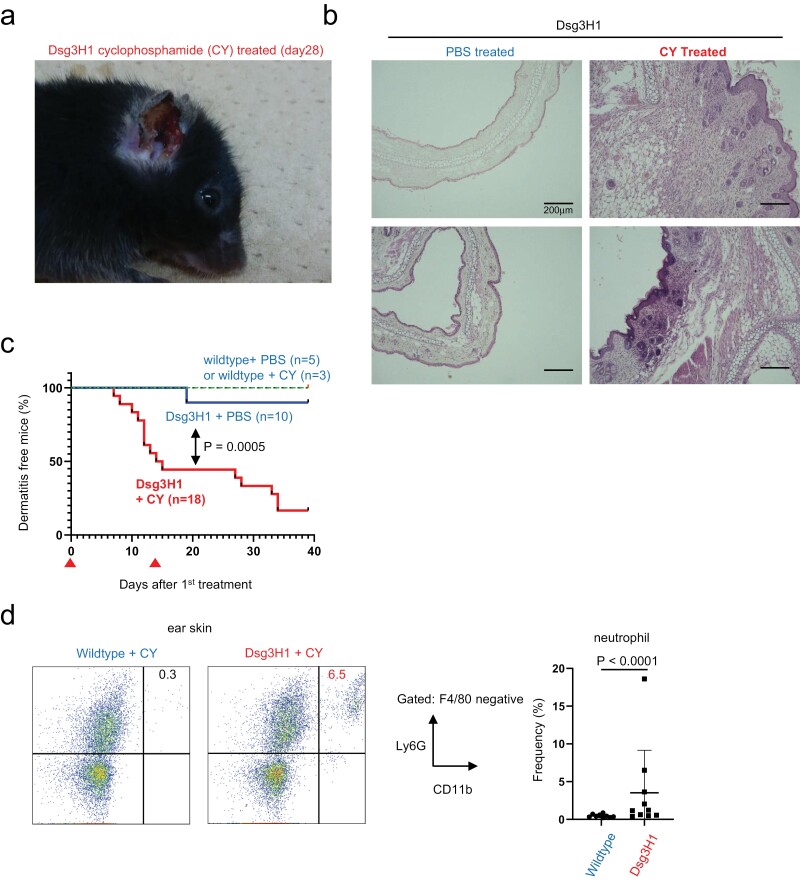
Cyclophosphamide (CY) treatment provokes massive skin inflammation in Dsg3H1 mice. Dsg3H1 mice were treated with CY (200 mg/kg body weight) or PBS. (a) Representative development of dermatitis. (b) Histology of ears. The scale bars: 200 mm. (c) Incidence of dermatitis. CY or PBS was injected intraperitoneally on days 0 and 14 (indicated by arrowheads ▲). Data from two independent experiments were combined. (Log-rank test, *P* < .0005). (d) Neutrophil infiltration in CY-treated mice (two-tailed *t*-test, mean ± SD).

TBI induces tissue damage in various tissues, which might have caused severe weight loss and death independently of LIP-induced immune activation. In search of another model of transient lymphopenia, we focused on CY, which is not only an anticancer agent but also depletes peripheral T cells. A non-myeloablative dose of CY is known to cause transient lymphopenia, followed by earlier onset and accelerated autoimmune diabetes in non-obese diabetic (NOD) mice (20, 21). We found that the same treatment induced slower than, but as strong, dermatitis as TBI in Dsg3H1Tg mice (Fig. 3a–c). Unlike TBI, CY did not induce death in the affected mice. The findings from TBI and CY together suggest that tolerance in Dsg3H1 mice is not absolute but can be broken by transient lymphopenia.

### Lymphopenia induces a Th17 response in DsgH1 mice

**Figure 4.**
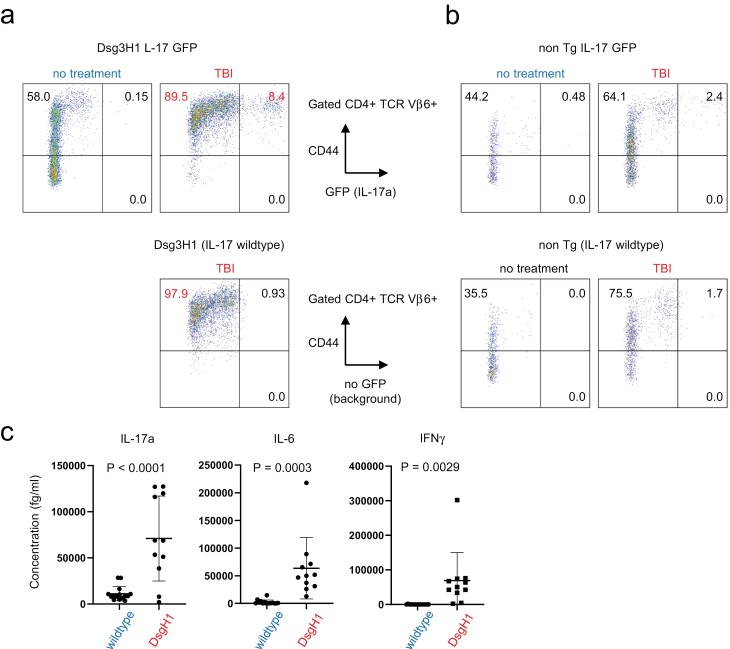
Induction of a Th17 response by transient lymphopenia in Dsg3H1 mice. (a) Th17 development in Dsg3H1 mice by TBI. Dsg3H1 mice are further bred to the IL-17-eGFP reporter strain and then subjected for TBI. 2 weeks later, cells from draining lymph nodes were analysed by FACS. (b) The data from non-TCR transgenic IL-17-eGFP reporter are shown (a, b two independent experiments including mice carrying each genotype and treatment). (c) SystemicTh17 response of CY-treated Dsg3H1 mice. Sera from CY-treated mice were measured with a high-sensitivity cytometric bead array. One representative set of data from two independent experiments (two-tailed *t*-test, mean ± SD).

In an adoptive transfer model, Th17-skewed pathogenic Dsg3H1 T cells cause neutrophilic inflammation in recipients (27, 28). Severe neutrophilic infiltrate was also observed in Dsg3H1 mice treated by TBI and CY, which was analogous to Th17-mediated pathology (Fig. 3d). Given that Th17 is a cause of neutrophilic inflammation (36, 37), we examined the involvement of Th17 cells in the current models. In Dsg3H1 mice bred to the IL-17-eGFP reporter strain, TBI induced upregulation of CD44 on most CD4^+^ TCR Vβ6^+^ cells, and a part became eGFP positive (Fig. 4a). Such upregulation of CD44 was milder on the non-TCR Tg counterpart (Fig. 4b). Since CD4^+^ TCR Vβ6^+^ cells contain a Tg-derived TCR, transient lymphopenia induced the rapid activation and differentiation of skin-reactive Th17 cells in Dsg3H1 mice *in vivo*. We also examined CY-treated Dsg3H1 mice. Massive induction of serum cytokines in these mice, but not in wildtype counterparts indicated a systemic Th17 response triggered by CY (Fig. 4c).

### Treg cells from Dsg3H1 mice were not affected by lymphopenia

**Figure 5.**
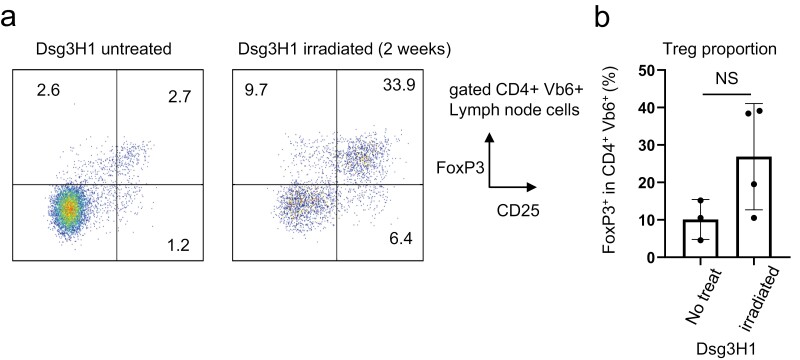
Treg cells from Dsg3H1 mice were not affected by lymphopenia. (a) FOXP3^+^ Treg cells from skin-draining lymph nodes of untreated, or irradiated Dsg3H1 mice were evaluated by FACS. (b) Summary of results. One representative set of data from two independent experiments is shown (two-tailed *t*-test, mean ± SD. NS, not significant).

FOXP3^**+**^ Treg cells play crucial roles in post-thymic tolerance. We reported that Treg cells mediate deletional tolerance when Dsg3H1 T cells were transferred in wildtype mice (26). Although our current model does not utilize adoptive transfer, we made several attempts to assess if FOXP3 + Treg were affected. We found that Treg cells did not show a decrease after TBI, but rather showed a tendency to increase their number (Fig. 5a and b). Tregs are known to undergo rapid LIP to recover their number (38, 39). Our data indicated the rapid repopulation of Treg cells; however, it did not prevent the activation of conventional skin-reactive T cells.

### Depletion of the intestinal microbiota prevents CY-induced dermatitis in Dsg3H1 mice

**Figure 6.**
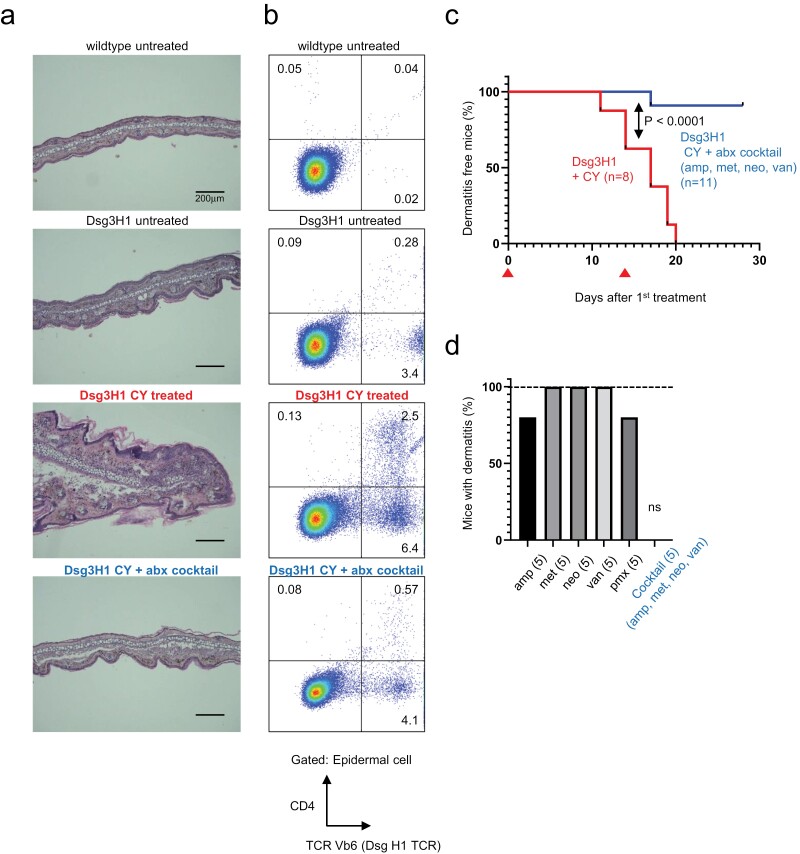
Broad-spectrum antibiotics prevent CY-induced dermatitis in Dsg3H1 mice. Mice were treated with CY with or without a cocktail of antibiotics (abx) containing ampicillin (amp), metronidazole (met), neomycin (neo), and vancomycin (van). (a) Representable microscopic images of an ear from mice. (b) Representative FACS of skin tissues. Note that TCR Vb6 + T cells are reduced by abx. (c) Cumulative incidence of dermatitis from two independent experiments. The arrowheads ▲ indicate the days of CY treatment (Log-rank test, *P* < .0001). (d) Incidence of dermatitis of Dsg3H1 mice treated by individual abx, or abx cocktail. The fraction of mice with dermatitis at day 30 was depicted (*n* = 5 each, ns, no symptoms observed in a group).

The commensal microbiota is an essential factor that influences T-cell responses. We noted that CY was reported to induce Th17 cells in a manner dependent on intestinal bacteria in a cancer model (40). To elucidate if microbiota is involved in the pathology of Dsg3H1 mice, we gave CY-treated mice a cocktail of four antibiotics (ampicillin, metronidazole, neomycin, and vancomycin), which is known to almost completely remove intestinal microbiota (41). We found that the treatment almost completely prevented CY-induced skin inflammation (Fig. 6a–c). Autoimmunity-associated Th17 is reported to be induced by Gram-positive bacteria that are sensitive to vancomycin (42, 43), an antibiotic specific for Gram-positive bacteria. In order to obtain information on the bacteria responsible in the current model, we treated mice with individual antibiotics. Unexpectedly, we found that none of each antibiotic, when administered alone, prevented CY-induced dermatitis (Fig. 6d), suggesting that variable intestinal microbiota, rather than specific species, was necessary for the development of CY-induced dermatitis in Dsg3H1 mice.

## Discussion

We demonstrated that a transient lymphopenia disrupts post-thymic tolerance in Dsg3H1 mice and causes dramatic skin symptoms. Although neonatal LIP drives polyclonal T cells to populate the periphery (15), LIP in adults favors the clonal expansion of T cells with a higher affinity to the self-MHC-antigen complex (44, 45). Indeed, both TBI and CY resulted in the selective expansion of Vβ6^+^ T cells derived from the DSG3-reactive TCR transgene. In addition, we found that the number of Treg cells was relatively unaffected, or rather showed an increase after lymphopenia. Treg cells are known to be resistant to TBI (38) or CY (39) and undergo rapid LIP to repopulate the lymphoid system. In wildtype C57BL/6 mice, Treg cells expand faster than conventional T cells after lymphopenia, that explains why this strain is generally resistant to spontaneous autoimmune diseases (18). Treg cells in Dsg3H1 mice (in the C57BL/6 background) normally underwent LIP, but it did not prevent autoimmune dermatitis. The data indicated that despite the increase in the number of Treg cells, moderately-to-highly autoreactive T cells abundant in these transgenic mice overcome control by Treg cells, leading to dermatitis. Furthermore, LIP induces T-cell differentiation into a CD44^high^, cytokine-positive effector/memory phenotype (15, 17). Therefore, LIP in adults contributes to autoimmunity by acting as a selective pressure favoring an oligoclonal expansion and activation of autoreactive T cells.

Treating Dsg3H1 mice with CY caused dermatitis only in the presence of intestinal microbiota. In previous studies investigating the relationship between the intestinal microbiota and LIP, Eri *et al*. (46) reported that treating nude mice with antibiotics inhibited both expansion and differentiation of transferred CD4^+^ donor cells. Similarly, it was suggested that commensal-dependent proliferation and generation of Th17 cells occur simultaneously in Rag2 KO mice (47). Unlike these studies, our model does not utilize adoptive transfer, however, based on these examples, it is likely that in our model, bacteria are responsible for both expansion and effector differentiation of autoreactive T cells. Regarding expansion, it was reported that germ-free mice lack rapid homeostatic proliferation in lymphopenic conditions (48). Other studies suggest that intestinal commensals stimulate IL-7 (49, 50), which primarily drives LIP (16, 51). With regards to differentiation, specific microbiota, such as segmented filamentous bacteria, are implicated in Th17-cell differentiation by inducing inflammatory genes (42, 43). However, vancomycin, which is effective against Gram-positive bacteria, alone failed to prevent CY-induced dermatitis development in Dsg3H1 Tg mice, suggesting the involvement of other Th17-inducing bacteria (52). It is intriguing to note that intestinal dysbiosis by lymphopenia drives T-cell activity. Both TBI and CY cause dysbiosis and bacterial translocation due to loss of integrity in the intestinal epithelium, which augments T-cell function (40, 53). TBI causes bacterial translocation, activating innate immune cells that prime strong expansion and activation of cytotoxic T cells in a cancer setting (53). CY also induces bacterial translocation that contributes to Th17 differentiation through Toll-like receptor-MYD88 mediated activation of innate immune cells (40, 54) and production of IL-6 (54). When this knowledge is interpreted together, our current model provides an essential interplay among lymphopenia, microbiota, and dysbiosis in the pathogenesis of skin autoimmunity.

In this study, we have established a novel, inducible skin autoimmune disease model in which the tolerance of defined Dsg3H1 T-cell is broken by TBI or CY treatment. Although CY has been known to induce LIP and accelerate islet-specific Th1-dependent autoimmunity in NOD mice (20, 21), a CY-dependent Th17-mediated skin disease model has not been previously reported. Presently animal models of chronic skin autoimmune disease largely rely on the topical application of imiquimod (55), cytokines (56), or adoptive transfer (25, 27, 28). Unlike many other models to examine the effect of LIP, our model does not require adoptive transfer into lymphopenic mice. Perhaps our current model also has significant implications in the pathology of actual autoimmune skin diseases. For example, idiopathic CD4 lymphocytopenia and HIV-induced immunodeficiency are associated with lymphopenia, dysbiosis, and autoimmunity, including skin diseases such as pemphigus and psoriasis (57–60). Additionally, acute viral infections such as coronavirus disease 2019 (COVID-19) can cause lymphopenia (61) and trigger psoriasis in some patients (62, 63). Notably, the presence of HLA Cw*0602, a risk allele of psoriasis, was present in 79% of HIV-positive patients with psoriasis, compared with 24.5% of HIV-infected patients without psoriasis (64), suggesting that lymphopenia due to HIV infection likely triggers expansion and differentiation of skin-reactive T cells with higher affinity to a particular MHC risk allele. Although this speculation is preliminary, our observations suggest a potential interplay between LIP caused by infection and the initiation of autoimmune skin diseases. Importantly, LIP appears to act as a trigger for autoimmunity, particularly in individuals with genetic predispositions. This insight underscores the complexity of autoimmune pathogenesis and emphasizes the need for a comprehensive understanding of the interplay between genetic and environmental factors in autoimmune diseases.

## References

[1] Palmer E. Negative selection—clearing out the bad apples from the T-cell repertoire. Nat Rev Immunol 2003;3:383–91. 10.1038/nri1085 12766760

[2] Haftmann C , Zwicky P, Ingelfinger F, et al. Protection against autoimmunity is driven by thymic epithelial cell-mediated regulation of T(reg) development. Sci Immunol 2021;6:eabf3111. 10.1126/sciimmunol.abf3111 34797691

[3] Brunkow ME , Jeffery EW, Hjerrild KA, et al. Disruption of a new forkhead/winged-helix protein, scurfin, results in the fatal lymphoproliferative disorder of the scurfy mouse. Nat Genet 2001;27:68–73. 10.1038/83784 11138001

[4] Wildin RS , Ramsdell F, Peake J, et al. X-linked neonatal diabetes mellitus, enteropathy and endocrinopathy syndrome is the human equivalent of mouse scurfy. Nat Genet 2001;27:18–20. 10.1038/83707 11137992

[5] Fontenot JD , Gavin MA, Rudensky AY. Foxp3 programs the development and function of CD4+ CD25+ regulatory T cells. Nat Immunol 2003;4:330–6. 10.1038/ni904 12612578

[6] Khattri R , Cox T, Yasayko SA, et al. An essential role for Scurfin in CD4+ CD25+ T regulatory cells. Nat Immunol 2003;4:337–42. 10.1038/ni909 12612581

[7] Wing K , Onishi Y, Prieto-Martin P, et al. CTLA-4 control over Foxp3+ regulatory T cell function. Science 2008;322:271–5. 10.1126/science.1160062 18845758

[8] Chinen T , Kannan AK, Levine AG, et al. An essential role for the IL-2 receptor in T(reg) cell function. Nat Immunol 2016;17:1322–33. 10.1038/ni.3540 27595233 PMC5071159

[9] Nishimura H , Nose M, Hiai H, et al. Development of lupus-like autoimmune diseases by disruption of the PD-1 gene encoding an ITIM motif-carrying immunoreceptor. Immunity 1999;11:141–51. 10.1016/s1074-7613(00)80089-8 10485649

[10] Hasegawa K , Martin F, Huang G, et al. PEST domain-enriched tyrosine phosphatase (PEP) regulation of effector/memory T cells. Science 2004;303:685–9. 10.1126/science.1092138 14752163

[11] Armitage LH , Wallet MA, Mathews CE. Influence of PTPN22 allotypes on innate and adaptive immune function in health and disease. Front Immunol 2021;12:636618. 10.3389/fimmu.2021.636618 33717184 PMC7946861

[12] Ogishi M , Yang R, Aytekin C, et al. Inherited PD-1 deficiency underlies tuberculosis and autoimmunity in a child. Nat Med 2021;27:1646–54. 10.1038/s41591-021-01388-5 34183838 PMC8446316

[13] Caudy AA , Reddy ST, Chatila T, et al. CD25 deficiency causes an immune dysregulation, polyendocrinopathy, enteropathy, X-linked-like syndrome, and defective IL-10 expression from CD4 lymphocytes. J Allergy Clin Immunol 2007;119:482–7. 10.1016/j.jaci.2006.10.007 17196245

[14] Egg D , Rump IC, Mitsuiki N, et al. Therapeutic options for CTLA-4 insufficiency. J Allergy Clin Immunol 2022;149:736–46. 10.1016/j.jaci.2021.04.039 34111452

[15] Min B , McHugh R, Sempowski GD, et al. Neonates support lymphopenia-induced proliferation. Immunity 2003;18:131–40. 10.1016/s1074-7613(02)00508-3 12530982

[16] Tan JT , Dudl E, LeRoy E, et al. IL-7 is critical for homeostatic proliferation and survival of naive T cells. Proc Natl Acad Sci USA 2001;98:8732–7. 10.1073/pnas.161126098 11447288 PMC37504

[17] Jameson SC. Maintaining the norm: T-cell homeostasis. Nat Rev Immunol 2002;2:547–56. 10.1038/nri853 12154374

[18] Le Campion A , Gagnerault MC, Auffray C, et al. Lymphopenia-induced spontaneous T-cell proliferation as a cofactor for autoimmune disease development. Blood 2009;114:1784–93. 10.1182/blood-2008-12-192120 19561321

[19] Bending D , De la Peña H, Veldhoen M, et al. Highly purified Th17 cells from BDC2.5NOD mice convert into Th1-like cells in NOD/SCID recipient mice. J Clin Invest 2009;119:565–72. 10.1172/JCI37865 19188681 PMC2648686

[20] Yasunami R , Bach JF. Anti-suppressor effect of cyclophosphamide on the development of spontaneous diabetes in NOD mice. Eur J Immunol 1988;18:481–4. 10.1002/eji.1830180325 2965652

[21] Harada M , Makino S. Promotion of spontaneous diabetes in non-obese diabetes-prone mice by cyclophosphamide. Diabetologia 1984;27:604–6. 10.1007/BF00276978 6530055

[22] Sprent J , Surh CD. Normal T cell homeostasis: the conversion of naive cells into memory-phenotype cells. Nat Immunol 2011;12:478–84. 10.1038/ni.2018 21739670 PMC3434123

[23] Takahashi H , Iriki H, Mukai M, et al. Autoimmunity and immunological tolerance in autoimmune bullous diseases. Int Immunol 2019;31:431–7. 10.1093/intimm/dxz030 30887049

[24] Takahashi H , Iriki H, Asahina Y. T cell autoimmunity and immune regulation to desmoglein 3, a pemphigus autoantigen. J Dermatol 2023;50:112–23. 10.1111/1346-8138.16663 36539957 PMC10107879

[25] Takahashi H , Kouno M, Nagao K, et al. Desmoglein 3-specific CD4+ T cells induce pemphigus vulgaris and interface dermatitis in mice. J Clin Invest 2011;121:3677–88. 10.1172/JCI57379 21821914 PMC3163963

[26] Iriki H , Takahashi H, Wada N, et al. Peripheral tolerance by Treg via constraining OX40 signal in autoreactive T cells against desmoglein 3, a target antigen in pemphigus. Proc Natl Acad Sci USA 2021;118:e2026763118. 10.1073/pnas.2026763118 34848535 PMC8670434

[27] Nishimoto S , Kotani H, Tsuruta S, et al. Th17 cells carrying TCR recognizing epidermal autoantigen induce psoriasis-like skin inflammation. J Immunol 2013;191:3065–72. 10.4049/jimmunol.1300348 23956432

[28] Tokifuji Y , Hayabuchi H, Sasaki T, et al. Targeting abatacept-resistant T-helper-17 cells by aldehyde dehydrogenase inhibition. iScience 2024;27:108646. 10.1016/j.isci.2023.108646 38226171 PMC10788227

[29] Cheung L , Weinstein M. Idiopathic CD4 T-cell lymphocytopenia: a case report of a young boy with recalcitrant warts. J Cutan Med Surg 2016;20:470–3. 10.1177/1203475416638045 26964553

[30] Staughton R. Skin manifestations in AIDS patients. Br J Clin Pract Suppl 1990;71:109–13. 2091731

[31] Baroudjian B , Viguier M, Battistella M, et al. Psoriasis associated with idiopathic CD4+ T-cell lymphopenia: a regulatory T-cell defect? Br J Dermatol 2014;171:186–9. 10.1111/bjd.12922 24579866

[32] Hardman CM , Baker BS, Lortan J, et al. Active psoriasis and profound CD4+ lymphocytopenia. Br J Dermatol 1997;136:930–2. 9217828

[33] Morar N , Willis-Owen SA, Maurer T, et al. HIV-associated psoriasis: pathogenesis, clinical features, and management. Lancet Infect Dis 2010;10:470–8. 10.1016/S1473-3099(10)70101-8 20610329

[34] Price AE , Reinhardt RL, Liang HE, et al. Marking and quantifying IL-17A-producing cells *in vivo*. PLoS One 2012;7:e39750. 10.1371/journal.pone.0039750 22768117 PMC3387253

[35] Bosco N , Swee LK, Bénard A, et al. Auto-reconstitution of the T-cell compartment by radioresistant hematopoietic cells following lethal irradiation and bone marrow transplantation. Exp Hematol 2010;38:222–32.e2. 10.1016/j.exphem.2009.12.006 20045443

[36] Littman DR , Rudensky AY. Th17 and regulatory T cells in mediating and restraining inflammation. Cell 2010;140:845–58. 10.1016/j.cell.2010.02.021 20303875

[37] Yasuda K , Takeuchi Y, Hirota K. The pathogenicity of Th17 cells in autoimmune diseases. Semin Immunopathol 2019;41:283–97. 10.1007/s00281-019-00733-8 30891627

[38] Komatsu N , Hori S. Full restoration of peripheral Foxp3+ regulatory T cell pool by radioresistant host cells in scurfy bone marrow chimeras. Proc Natl Acad Sci USA 2007;104:8959–64. 10.1073/pnas.0702004104 17494743 PMC1885610

[39] Kanakry CG , Ganguly S, Zahurak M, et al. Aldehyde dehydrogenase expression drives human regulatory T cell resistance to post-transplantation cyclophosphamide. Sci Transl Med 2013;5:211ra157. 10.1126/scitranslmed.3006960 PMC4155575 24225944

[40] Viaud S , Saccheri F, Mignot G, et al. The intestinal microbiota modulates the anticancer immune effects of cyclophosphamide. Science 2013;342:971–6. 10.1126/science.1240537 24264990 PMC4048947

[41] Rakoff-Nahoum S , Paglino J, Eslami-Varzaneh F, et al. Recognition of commensal microflora by toll-like receptors is required for intestinal homeostasis. Cell 2004;118:229–41. 10.1016/j.cell.2004.07.002 15260992

[42] Ivanov II , Frutos Rde L, Manel N, et al. Specific microbiota direct the differentiation of IL-17-producing T-helper cells in the mucosa of the small intestine. Cell Host Microbe 2008;4:337. 18854238 10.1016/j.chom.2008.09.009 PMC2597589

[43] Ivanov II , Atarashi K, Manel N, et al. Induction of intestinal Th17 cells by segmented filamentous bacteria. Cell 2009;139:485–98. 10.1016/j.cell.2009.09.033 19836068 PMC2796826

[44] Kassiotis G , Zamoyska R, Stockinger B. Involvement of avidity for major histocompatibility complex in homeostasis of naive and memory T cells. J Exp Med 2003;197:1007–16. 10.1084/jem.20021812 12707300 PMC2193871

[45] Kieper WC , Burghardt JT, Surh CD. A role for TCR affinity in regulating naive T cell homeostasis. J Immunol 2004;172:40–4. 10.4049/jimmunol.172.1.40 14688307

[46] Eri T , Kawahata K, Kanzaki T, et al. Intestinal microbiota link lymphopenia to murine autoimmunity via PD-1(+)CXCR5(−/dim) B-helper T cell induction. Sci Rep 2017;7:46037. 10.1038/srep46037 28443628 PMC5405410

[47] Kawabe T , Sun SL, Fujita T, et al. Homeostatic proliferation of naive CD4+ T cells in mesenteric lymph nodes generates gut-tropic Th17 cells. J Immunol 2013;190:5788–98. 10.4049/jimmunol.1203111 23610141

[48] Kieper WC , Troy A, Burghardt JT, et al. Recent immune status determines the source of antigens that drive homeostatic T cell expansion. J Immunol 2005;174:3158–63. 10.4049/jimmunol.174.6.3158 15749843

[49] Shalapour S , Deiser K, Sercan O, et al. Commensal microflora and interferon-gamma promote steady-state interleukin-7 production *in vivo*. Eur J Immunol 2010;40:2391–400. 10.1002/eji.201040441 20690180

[50] Shalapour S , Deiser K, Kühl AA, et al. Interleukin-7 links T lymphocyte and intestinal epithelial cell homeostasis. PLoS One 2012;7:e31939. 10.1371/journal.pone.0031939 22384106 PMC3288069

[51] Guimond M , Veenstra RG, Grindler DJ, et al. Interleukin 7 signaling in dendritic cells regulates the homeostatic proliferation and niche size of CD4+ T cells. Nat Immunol 2009;10:149–57. 10.1038/ni.1695 19136960 PMC2713006

[52] Atarashi K , Tanoue T, Ando M, et al. Th17 cell induction by adhesion of microbes to intestinal epithelial cells. Cell 2015;163:367–80. 10.1016/j.cell.2015.08.058 26411289 PMC4765954

[53] Paulos CM , Wrzesinski C, Kaiser A, et al. Microbial translocation augments the function of adoptively transferred self/tumor-specific CD8+ T cells via TLR4 signaling. J Clin Invest 2007;117:2197–204. 10.1172/JCI32205 17657310 PMC1924500

[54] Feng T , Wang L, Schoeb TR, et al. Microbiota innate stimulation is a prerequisite for T cell spontaneous proliferation and induction of experimental colitis. J Exp Med 2010;207:1321–32. 10.1084/jem.20092253 20498021 PMC2882839

[55] van der Fits L , Mourits S, Voerman JS, et al. Imiquimod-induced psoriasis-like skin inflammation in mice is mediated via the IL-23/IL-17 axis. *J Immunol* 2009;182:5836–45. 10.4049/jimmunol.0802999 19380832

[56] Chan JR , Blumenschein W, Murphy E, et al. IL-23 stimulates epidermal hyperplasia via TNF and IL-20R2-dependent mechanisms with implications for psoriasis pathogenesis. *J Exp Med* 2006;203:2577–87. 10.1084/jem.20060244 17074928 PMC2118145

[57] Zhang X , Shi L, Sun T, et al. Dysbiosis of gut microbiota and its correlation with dysregulation of cytokines in psoriasis patients. BMC Microbiol 2021;21:78. 10.1186/s12866-021-02125-1 33685393 PMC7941898

[58] Hidalgo-Cantabrana C , Gómez J, Delgado S, et al. Gut microbiota dysbiosis in a cohort of patients with psoriasis. Br J Dermatol 2019;181:1287–95. 10.1111/bjd.17931 30920647

[59] Wang Y , Xia X, Zhou X, et al. Association of gut microbiome and metabolites with onset and treatment response of patients with pemphigus vulgaris. Front Immunol 2023;14:1114586. 10.3389/fimmu.2023.1114586 37122759 PMC10140300

[60] Han Z , Fan Y, Wu Q, et al. Comparison of gut microbiota dysbiosis between pemphigus vulgaris and bullous pemphigoid. Int Immunopharmacol 2024;128:111470. 10.1016/j.intimp.2023.111470 38185033

[61] Fathi N , Rezaei N. Lymphopenia in COVID-19: therapeutic opportunities. Cell Biol Int 2020;44:1792–7. 10.1002/cbin.11403 32458561 PMC7283672

[62] Rouai M , Rabhi F, Mansouri N, et al. New-onset guttate psoriasis secondary to COVID-19. *Clin Case Rep* 2021;9:e04542. 10.1002/ccr3.4542 34336212 PMC8319378

[63] Mathieu RJ , Cobb CBC, Telang GH, et al. New-onset pustular psoriasis in the setting of severe acute respiratory syndrome coronavirus 2 infection causing coronavirus disease 2019. *JAAD Case Rep* 2020;6:1360–2. 10.1016/j.jdcr.2020.10.013 33102669 PMC7566789

[64] Mallon E , Young S, Bunce M, et al. HLA-Cw*0602 and HIV-associated psoriasis. *Br J Dermatol* 1998;139:527–33. 10.1046/j.1365-2133.1998.02495.x 9767306

